# Metabolic syndrome as a common comorbidity in adults with hypothalamic dysfunction

**DOI:** 10.3389/fendo.2022.973299

**Published:** 2022-10-13

**Authors:** Zhuoran Xu, Xiaoan Ke, Xianxian Yuan, Linjie Wang, Lian Duan, Yong Yao, Kan Deng, Feng Feng, Hui You, Xin Lian, Renzhi Wang, Hongbo Yang, Hui Pan, Lin Lu, Huijuan Zhu

**Affiliations:** ^1^ Department of Endocrinology, State Key Laboratory of Complex Severe and Rare Diseases, The Translational Medicine Center of Peking Union Medical College Hospital (PUMCH), PUMCH, Chinese Academy of Medical Sciences and Peking Union Medical College (CAMS & PUMC), Beijing, China; ^2^ Key Laboratory of Endocrinology of National Health Commission of the People’s Republic of China, The Translational Medicine Center of Peking Union Medical College Hospital (PUMCH), PUMCH, Chinese Academy of Medical Sciences and Peking Union Medical College (CAMS & PUMC), Beijing, China; ^3^ Division of Endocrinology and Metabolism, Department of Obstetrics, Beijing Obstetrics and Gynecology Hospital, Capital Medical University, Beijing, China; ^4^ Department of Neurosurgery, Peking Union Medical College Hospital, Beijing, China; ^5^ Department of Radiology, Peking Union Medical College Hospital, Beijing, China; ^6^ Department of Radiation Oncology, Peking Union Medical College Hospital, Beijing, China

**Keywords:** hypothalamic dysfunction, hypothalamic obesity, metabolic syndrome, metabolic comorbidities, hypopituitarism, craniopharyngioma

## Abstract

**Objective:**

Hypothalamic dysfunction (HD) results in various endocrine disorders and is associated with an increased risk of metabolic comorbidities. This study aimed to analyze the clinical characteristics and metabolic abnormalities of adults with HD of various causes.

**Methods:**

This study retrospectively reviewed adults with HD treated at our center between August 1989 and October 2020. Metabolic characteristics of patients were compared to those of age-, sex-matched lean, and body mass index (BMI)-matched controls.

**Results:**

Temperature dysregulation (61.0%) was the most common hypothalamic physiological dysfunction. At least one anterior pituitary hormone deficiency was observed in 50 patients (84.7%), with hypogonadotropic hypogonadism being the most frequently observed. Metabolic syndrome was confirmed in 31 patients (52.5%) and was significantly more prevalent in those with panhypopituitarism or overweight/obesity. Metabolic syndrome (MetS) was significantly more common in patients with HD than in both lean and BMI-matched controls (P < 0.001 and P = 0.030, respectively). Considering the components of MetS, elevated fasting glucose levels were significantly more common in patients with HD than in BMI-matched controls (P = 0.029). Overweight/obesity and panhypopituitarism were significant risk factors for MetS in the multivariate analysis on patients with HD. Moreover, in the multivariate analysis on patients and BMI-matched control, HD was a significant risk factor of MetS (P=0.035, OR 2.919) after adjusted for age, sex and BMI.

**Conclusions:**

Temperature dysregulation and hypogonadotropic hypogonadism are the most common physiological and endocrine dysfunctions, respectively. MetS and unfavorable metabolic profiles were prevalent in adults with HD. HD was a significant risk factor of MetS after adjusted for BMI.

## Introduction

The hypothalamus is an ancient brain structure known to orchestrate complex physiological responses to maintain internal homeostasis (1). Damage to the hypothalamus results in various endocrine, metabolic, and neurologic disorders, usually referred to as hypothalamic dysfunction (HD) (2). Patients with hypothalamic lesions exhibit symptoms of hypothalamic physiological dysfunction such as alterations in satiety, thirst response, circadian rhythms, autonomic dysregulation, and endocrine dysfunction secondary to hypothalamic impairment (3).

Previous studies demonstrated that HD-related symptoms contribute to impaired quality of life in patients with suprasellar tumors (4, 5). In addition, damage to the hypothalamic-pituitary axis is associated with metabolic comorbidities, including abdominal obesity, elevated cholesterol/triglyceride, and insulin resistance (6, 7), and further leads to an increased risk of cardiovascular diseases and poor long-term prognosis. However, pronounced heterogeneity in etiology and clinical features leads to difficulties in developing a handy guideline for the accurate diagnosis and proper treatment of HD.

Most published studies have focused on children and adolescents with HD in which polydipsia/polyuria, obesity, and eating disorders are typical symptoms (3). However, the causes and clinical characteristics of HD in adults have not been systematically documented. Moreover, although hypothalamic involvement has been associated with an increased risk of metabolic disorders in patients with craniopharyngioma (8), the prevalence of metabolic comorbidities and their risk factors in adults with HD has not been systematically documented. Therefore, in the current study, we aimed to investigate the causes and clinical features of HD and examine the prevalence of metabolic syndrome and its influencing factors in adults with HD.

## Patients and methods

### Patients

We retrospectively reviewed patients aged > 18 years diagnosed with HD at our center between August 1989 and October 2020. Controls were asymptomatic patients randomly selected from the obesity clinic and health checkup records between 2009 and 2010. Case-and body mass index (BMI)-matched controls were 1:1 matched for age, sex, and BMI. Lean control (whose BMI ≥ 18.0 kg/m^2^ and<24.0 kg/m^2^) was 1:1 matched in the case group by age and sex. The studies involving human participants were reviewed and approved by the Ethics Committee of the Peking Union Medical College Hospital (PUMCH). The patients provided their written informed consent to participate in this study.

### Definitions

No clinical diagnostic criteria have been generally accepted for HD in adults as far as we are concerned. In this study, the clinical cases should match the following definition of HD (1): hypothalamic physiological dysfunction, which means showing at least two of the following clinical signs that cannot be fully explained by other diseases: rapid weight gain, voracious appetite/anorexia, adipsia, electrolyte abnormalities, behavioral abnormalities, and autonomic dysregulation (e.g., thermal dysregulation, bradycardia, strabismus, sweating alteration, and digestive dysmotility) (2) endocrine dysfunction not fully explained by a single target gland or simple pituitary damage (3) diabetes insipidus (3, 9). Cases were suspected of HD if they present with criteria (1) and at least one item in criteria (2) or (3). Diagnoses were prudently made by experienced endocrinologists and reviewed by a multidisciplinary team. Patients were screened for possible causes of HD after admission or were defined as idiopathic, excluding other causes.

The diagnosis of eating disorders, including binge eating disorders and anorexia, was based on the Diagnostic and Statistical Manual of Mental Disorders, Fifth Edition (DSM-5) (10). Hypersomnia was screened using the Epworth Sleepiness Scale (ESS) (11). Central fever was defined as a body temperature above 37.3 °C after excluding infectious fever. The criteria included (1): no culture from a normally sterile source growing a pathogenic species; and (2) no documented clinical diagnosis of infection and sepsis syndrome, with negligible therapeutic effects of antibiotics (12). Hypothermia was defined as a body temperature below 35°C after excluding infectious factors, drug-induced hypothermia, and other metabolic disorders (13). Patients whose body temperature fluctuated >2°C in a week and changed along with ambient temperature were described as difficulty in maintain a constant body temperature. Abnormal sweating was identified based on patients’ self-reports of their frequency of excessive sweating, sweating at night, less sweating in hot weather, and asymmetric differences in sweating.

Growth hormone deficiency (GHD) was considered under the following conditions: (1) low serum insulin-like growth factor 1 (IGF-1) concentration in the presence of three other pituitary hormone deficiencies or (2) low peak GH value in the GH stimulation tests (14, 15). Central hypothyroidism was diagnosed based on decreased fT4 and low, normal, or mildly elevated thyroid-stimulating hormone (TSH) levels in the absence of non-thyroidal illness (14, 15). Central adrenal insufficiency (CAI) was diagnosed when serum cortisol level at 8 am<3 μg/dL. Patients with a serum cortisol level between 3 and 18 μg/dL underwent an insulin-induced hypoglycemia test and were confirmed to have CAI when the peak cortisol level was<18 μg/dL (16). Hypogonadotropic hypogonadism (HH) was confirmed in men with low morning serum total testosterone (<10.4 nmol/L) and in women with non-elevated luteinizing hormone (LH) and follicle-stimulating hormone (FSH) levels in the presence of oligomenorrhea or amenorrhea (15, 17). Panhypopituitarism was confirmed with three or more anterior pituitary hormone deficiencies (18). Central diabetes insipidus (CDI) was diagnosed in patients with polyuria if serum osmolarity>295 mOsm/kg while urine osmolarity was<300 mOsm/kg after water deprivation and urine osmolarity increased > 50% following desmopressin (19). Thirst sensation was evaluated using the visual analog scale (VAS). Adipsic diabetes insipidus was considered if patients demonstrated subnormal thirst responses to hypertonic saline, with a water intake of less than half of the lower limit of normal (< 500 mL) (20).

Metabolic syndrome (MetS) was diagnosed according to the National Cholesterol Education Program Adult Treatment Panel III (NCEP/ATP-III) and modified to cater to the Asian population (21, 22). At least three of the following criteria were met for the diagnosis of MetS: obesity (waist circumference [WC] ≥ 90 cm in males and ≥ 80 cm in females, or BMI ≥ 25 kg/m^2^ in the Chinese population), elevated triglycerides (≥ 150 mg/dL or ongoing anti-hyperlipidemia treatment), reduced high-density lipoprotein-cholesterol (HDL-C) (< 40 mg/dL in men, < 50 mg/dL in women or ongoing anti-hyperlipidemia treatment), hypertension (blood pressure ≥ 130/85 mmHg or ongoing antihypertensive treatment), and elevated fasting glucose (fasting glucose ≥ 110 mg/dL or previously diagnosed with type 2 diabetes mellitus).

### Data collection

Demographic characteristics, anthropometric data, and clinical features, including symptoms suggestive of pituitary deficiency and other neuroendocrine involvement, were extracted from medical records. Biochemical tests and pituitary function evaluations were completed within 3 days after the first admission. In addition, abdominal enhanced computed tomography (CT) and ultrasound were conducted to confirm the diagnosis of nonalcoholic fatty liver disease (NAFLD), and magnetic resonance imaging (MRI) was performed for the evaluation of pituitary and hypothalamic involvement.

### Statistical analysis

The data analysis was performed using Statistical Package for the Social Sciences (SPSS) version 26.0 (SPSS Inc., Chicago, IL, USA). The results were presented as medians with interquartile ranges for descriptive data and proportions for categorical variables. Univariate analysis for numerical variables were performed using the Mann–Whitney U test or Wilcoxon signed-rank test, whereas chi-square tests or Fisher’s exact test were performed for categorical variables. Multivariate analysis was performed with logistic regression (forward: LR). All data were considered to be statistically significant at P < 0.05.

## Results

### Demographic and clinical characteristics

The final study population comprised 59 patients; 27 were male (45.8%), and 32 (54.2%) were female. The demographic and clinical characteristics of the participants are presented in **Table 1**. The median age at diagnosis was 33.9 (range, 18.0 to 74.5). The median BMI at diagnosis was 26.5 kg/m^2^ (range, 15.1 to 53.3 kg/m^2^).

**Table 1:** Demographic, clinical and biochemical characteristics of adults with hypothalamic dysfunction (n=59).

	Median (IQRs)
Demographic characteristics	
Male, %	27 (45.8%)
Age, years	33.9 (24.9, 48.8)
Clinical characteristics	
BMI, kg/m^2^	26.5 (22.8, 30.9)
SBP, mmHg	114 (100, 125)
DBP, mmHg	73 (65, 80)
Biochemical characteristics	
GLU, mmol/L	4.8 (4.5, 5.7)
F-INS,μIU/mL	26.5 (13.6, 46.7)
HbA1c%	5.9 (5.3, 8.7)
TC, mmol/L	4.98 (3.96, 5.78)
TG, mmol/L	1.65 (1.05, 3.02)
HDL-C,mmol/L (male)	0.94 (0.72, 1.34)
HDL-C, mmol/L(female)	0.91 (0.67, 1.26)
LDL-C, mmol/L	2.99 (2.40, 3.54)
UA, μmol/L	363 (250, 447)

Data given as median (interquartile range) unless otherwise stated.

BMI, body mass index; SBP, systolic blood pressure; DBP, diastolic blood pressure; GLU, plasma glucose; F-INS, fasting insulin; HbA1c, glycated hemoglobin A1C; TC, total cholesterol; TG, triglycerides; HDL-C, high density lipoprotein cholesterol; LDL-C, low density lipoprotein cholesterol; UA, uric acid.

**Figure 1:**
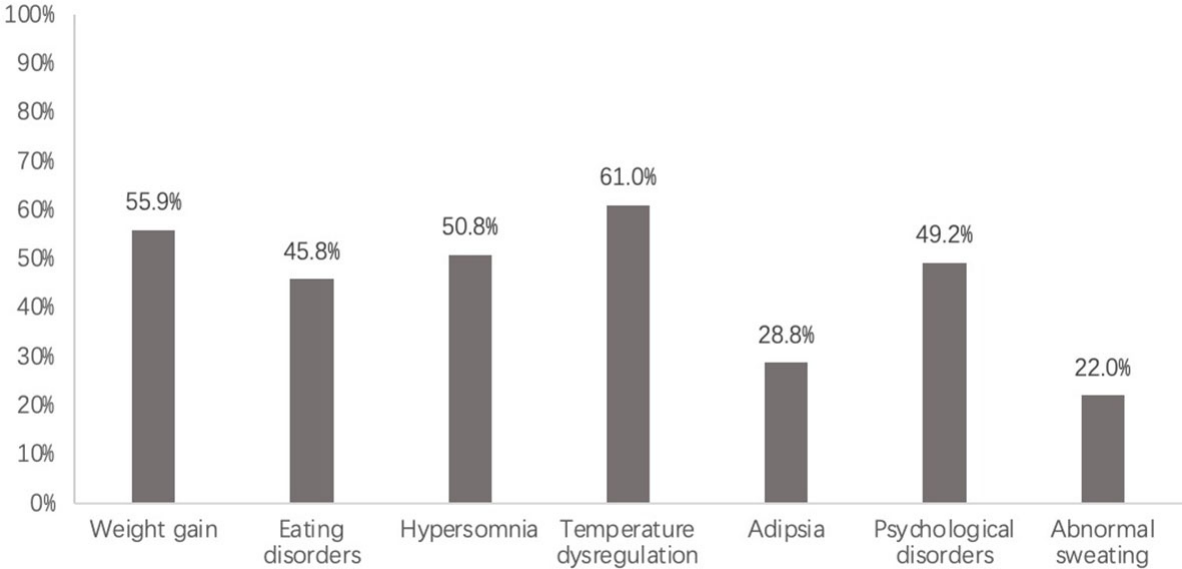
Clinical symptoms of adults with hypothalamic dysfunction. Data given at no. (%).

Temperature dysregulation (36 cases, 61.0%) was the most common hypothalamic physiological dysfunction observed in our study. Out of 36 cases, 33 showed constant or intermittent central fever, 2 showed hypothermia, and 1 showed difficulty maintaining a constant body temperature. Weight gain (33 cases, 55.9%) and hypersomnia (30 cases, 50.8%) were also common, followed by psychological disorders (29 cases, 49.2%), eating disorders (27 cases, 45.8%), adipsia (17 cases, 28.8%), and abnormal sweating (13 cases, 22.0%) **Figure 1**. Among the 33 patients presented with weight gain, 16 were hyperphagic, 15 showed no evidence of eating disorders, and 2 presented with anorexia. The rate of weight gain varied extensively, ranging from a steady increase of 2.5 kg/year to a rapid increase of 18 kg/month. The median weight gain rate was 12 kg/year.

### Endocrine assessment

CDI was a common endocrinopathy, confirmed in 48 patients (81.4%). Their median serum sodium level was 147.0 mmol/L (range, 133 to 168 mmol/L) upon admission. Adipsic diabetes insipidus (ADI) was diagnosed in 16 of 48 patients. The median serum sodium level of patients with ADI was 150.5 mmol/L (range, 134–168 mmol/L) upon admission, higher than that of patients with CDI with an intact thirst sensation (144 mmol/L). However, no statistically significant difference was noted (P = 0.066).

At least one anterior pituitary hormone deficiency was confirmed in 50 (84.7%) patients. Hypogonadotropic hypogonadism, detected in 22 men and 27 women, was the most common anterior pituitary dysfunction observed in our study (**Table 2**). Central hypothyroidism (45 cases, 76.3%) occurred second most frequently, followed by CAI (40 cases, 67.8%) and GHD (33 cases, 55.9%). Complete evaluation of the anterior pituitary function was performed in 39 patients, and 32 of 39 (82.1%) were diagnosed with panhypopituitarism.

**Table 2:** Hypothalamic-pituitary endocrine dysfunction of adults with hypothalamic dysfunction (n=59).

	GH Axis	HPG Axis	HPT Axis	HPA Axis	CDI
**Impaired**	33 (55.9%)	49 (83.0%)	45 (76.3%)	40 (67.8%)	48 (81.4%)
**Intact**	7 (11.9%)	5 (8.5%)	10 (16.9%)	14 (23.7%)	10 (16.9%)
**Not evaluated**	19 (32.2%)	5 (8.5%)	4 (6.8%)	5 (8.5%)	1 (1.7%)

Data given as no. (%). GH, growth hormone; HPG, hypothalamus-pituitary-gonadal; HPT, hypothalamus-pituitary-thyroid; HPA, hypothalamus-pituitary-adrenal; CDI, central diabetes insipidus.

### Etiology

We classified the etiology of all patients into natural onset and iatrogenic onset (**Table 3**). A total of 35 patients (59.3%) developed HD before receiving any iatrogenic intervention. The most common cause of HD with natural onset was intracranial neoplasm (28 cases, 47.5%), with germ cell tumors serving as the most common pathological diagnosis. Three patients developed HD due to primary or metastatic lymphoma, and 11 presented with masses in the hypothalamus region without a confirmed pathological diagnosis.

**Table 3:** Causes of hypothalamic dysfunction (n=59).

	*n* (%)
*Natural Onset*	35 (59.3%)
Neoplasm	28 (47.5%)
* Solid tumor*	
Pituitary adenoma	3
Germ cell tumor	7
Langerhans cell histiocytosis	3
Spindle cell carcinoma	1
* Hematologic tumor*	
Metastatic non-Hodgkin lymphoma	2
Diffuse large B cell lymphoma	1
* Unidentified mass*	11
Inflammation * Infectious*	4 (6.8%)
Viral encephalitis	1
* Autoimmune*	
Lymphadenitis granulomatosis	1
Lymphocytic hypophysitis	2
Congenital abnormality	3 (5.1%)
Rathke cyst	2
Empty sellar	1
*Iatrogenic Onset*	18 (30.5%)
Post-surgery	13 (22.0%)
Craniopharyngioma*	7
Pituitary adenoma	3
Meningioma	1
Chromophobe adenoma	1
Chronic hypothalamitis	1
Post-radiotherapy	5 (8.5%)
Langerhans cell histiocytosis	2
Unidentified mass	3
Unknown	6 (10.2%)

Data given as no. (%).

*1 patient went through a course of radiotherapy between two surgeries and developed signs of HTS within 1 month after the second surgery.

Eighteen patients (30.5%) developed HD after surgery or radiotherapy. The median time from treatment to metabolic evaluation was 5.50 months (P25: 1.75 months, P75: 23.50 months). Seven patients developed HD after craniopharyngioma resection and three after pituitary adenoma resection. Despite the 3 patients whose disease onset was not clearly noted, all 10 patients exhibited at least one symptom of HD within 1 month after surgery. Five patients presented with HD after radiotherapy, all of whom received gamma knife treatment. The interval between radiotherapy initiation and HD varied, ranging from 1 month to 1 year.

The etiology of HD was not clearly identified in six patients. One patient was diagnosed with a sellar cyst but showed no remission after two surgeries. One patient was suspected of having a diffuse hypothalamic lesion that was heterogeneously enhanced on MRI, but she was not capable of undergoing a subsequent biopsy. Four patients underwent etiology screening, and no signs were indicative of possible causes.

### Metabolic sequela

The prevalence of metabolic sequelae is shown in **Table 4**. A total of 56 cases (94.9%) had at least one metabolic complication. Dyslipidemia (50 cases, 84.7%) occurred most frequently, with reduced HDL-C level (34 cases, 57.6%) being the most common clinical type. Overweight/obesity (34 cases, 57.6%) and NAFLD (27 cases, 45.8%) were also common, whereas impaired glucose metabolism, hyperuricemia, and hypertension occurred less frequently.

**Table 4:** Metabolic sequela of adults with hypothalamic dysfunction (*n*=59).

Metabolic sequela	*n* (%)	Metabolic sequela	*n (%)*
Overweight/Obesity	34 (57.6%)	Metabolic syndrome	31 (52.5%)
Dyslipidemia	50 (84.7%)	Central obesity	31 (52.5%)
Diabetes		Elevated triglycerides	26 (44.1%)
IR	6 (10.2%)	Reduced HDL-C	34 (57.6%)
IFG/IGT	7 (11.9%)	Hypertension	18 (30.5%)
DM	16 (27.1%)	Elevated fasting glucose	20 (33.9%)
Hyperuricemia	21 (35.6%)	NAFLD	27 (45.8%)

Data given as no. (%).

IFG, impaired fasting glucose; IGT, impaired glucose tolerance; IR, insulin resistance; DM, diabetes mellitus; HDL-C, high density lipoprotein cholesterol; NAFLD, nonalcoholic fatty liver disease.

MetS was confirmed in 31 patients (52.5%). Considering its components, reduced HDL-C levels (34 cases, 57.6%) and obesity (31 cases, 52.5%) were comparatively common. In the univariate analysis, we found a significantly higher prevalence of overweight/obesity and psychological disorders in patients with MetS(P=0.007, P=0.050, respectively), while temperature dysregulation, hypersomnia and visual defects were not significantly correlated with MetS. Panhypopituitarism was also significantly more prevalent in patients with MetS (P=0.035), while no single axis deficiency (GHD, hypogonadotropic hypogonadism, central hypothyroidism, CAI and CDI) was significantly correlated with MetS. Age, sex and factors significantly correlated with MetS in the univariate analysis were included in the multivariate analysis (shown in **Table 5**). The results indicated that only overweight/obesity and panhypopituitarism were independent risk factors of MetS (P=0.005, P=0.040, respectively).

**Table 5:** Univariate and multivariate predictors of metabolic syndrome in adults with hypothalamic dysfunction.

	Patients with MetS	Patients without MetS	Univariate	Multivariate	
	(n=31)	(n=28)	P value	P value	OR (95% CI)
Age	33.9 (25.5, 46.2)	33.6 (24.0, 48.2)	0.939	0.204	
Sex					
Male	14	13	0.922	0.337	
Symptoms					
Overweight/Obesity	23	11	0.007	0.005	10.038 (2.007, 50.203)
Hypersomnia	14	16	0.358		
Temperature dysregulation	17	19	0.306		
Psychological disorders	19	10	0.050	0.057	
Visual defects	14	15	0.519		
Endocrine dysfunction					
GH axis	19/21	14/19	0.163		
HPG axis	27/29	22/25	0.653		
HPT axis	26/29	19/26	0.164		
HPA axis	25/30	15/24	0.120		
CDI	27/31	21/27	0.489		
Panhypopituitarism	20/21	12/18	0.035	0.040	13.481 (1.129, 160.985)

MetS, metabolic syndrome; OR, odd ratio; CI, confidence interval; GH, growth hormone; HPG, hypothalamus-pituitary-gonadal; HPT, hypothalamus-pituitary-thyroid; HPA, hypothalamus-pituitary-adrenal; CDI, central diabetes insipidus.

We then analyzed the metabolic characteristics of patients with HD in comparison with age-, sex-matched lean control, age-, sex-, and BMI-matched control patients. **Table 6** presents the results. WC was measured in 17 patients undergoing HD. The median WC was 107.0 cm (range, 61.0 to 143.0 cm), which was significantly higher than that of the lean control group (P = 0.001) and higher than that of the BMI-matched control, although not statistically significant (P = 0.061). MetS was significantly more common in patients with HD than those in the lean and BMI-matched controls (P < 0.001 and P = 0.030, respectively). With regard to the components of MetS, we found a significantly higher prevalence of elevated triglycerides, elevated fasting glucose, and central obesity in patients with HD than those in the lean control group (P = 0.045, P = 0.003, and P < 0.001, respectively). In addition, compared with BMI-matched controls, a significantly higher prevalence of elevated fasting glucose (P = 0.029) was observed in patients with HD.

**Table 6:** Metabolic diseases and biochemical characteristics of adults with hypothalamic dysfunction in comparison with lean control and BMI-matched control (*n*=44).

	HD	Lean control	BMI-matched control	p value		
				HD vs Lean control	HD vs BMI-matched control	Lean vs BMI-matched control
Demographic characteristics						
Male, %	21 (47.8%)	21 (47.8%)	21 (47.8%)	/	/	/
Age, years	32.1 (24.8, 39.1)	31.5 (25.3, 38.8)	30.5 (26.0, 40.5)	0.119	0.572	0.807
Clinical characteristics						
BMI, kg/m^2^	26.9 (23.8, 30.9)	21.8 (20.3, 23.2)	27.3 (23.3, 30.9)	<0.001	0.744	<0.001
WC, cm (*n*=17)	107.0 (92.5, 111.0)	76.2 (72.3, 80.9)	91.0 (79.0, 97.0)	<0.001	0.061	<0.001
Metabolic syndrome						
MetS	23 (52.3%)	2 (4.5%)	13 (29.5%)	<0.001***	0.030*	0.003**
Hypertension, %	11 (25.0%)	7 (15.9%)	17 (38.6%)	0.290	0.170	0.017*
Elevated TG, %	20 (45.5%)	11 (25.0%)	16 (36.4%)	0.045*	0.386	0.248
Reduced HDL-C, %	26 (59.1%)	17 (38.6%)	20 (45.5%)	0.055	0.200	0.517
Elevated fasting glucose, %	13 (29.5%)	2 (4.5%)	4 (9.1%)	0.003**	0.029*	0.676
Central obesity, %	30 (68.2%)	7 (15.9%)	34 (77.3%)	<0.001***	0.338	<0.001***
Biochemical characteristics						
ALT, U/L	26.0 (17.0,54.0)	16.0 (11.0, 25.8)	24.0 (16.8, 43.0)	0.001***	0.260	0.006**
AST, U/L	35.0 (23.0, 52.0)	17.0 (15.0, 21.0)	21.5 (16.8, 28.3)	<0.001***	0.006**	0.017*
GLU, mmol/L	4.85 (4.50, 5.68)	5.20 (4.80, 5.70)	5.30 (5.00, 5.70)	0.851	0.368	0.517
TC, mmol/L	4.97 (3.76, 5.67)	3.89 (3.56, 4.54)	4.47 (4.04, 5.45)	0.004**	0.965	0.006**
TG, mmol/L	1.79 (1.07, 3.01)	0.99 (0.78, 1.68)	1.46 (0.92, 2.49)	0.002**	0.637	0.008**
HDL-C,mmol/L (male)	0.94 (0.71, 1.36)	1.24 (1.10, 1.41)	1.17 (0.94, 1.35)	0.054	0.133	0.421
HDL-C, mmol/L (female)	0.86 (0.67, 1.17)	1.26 (1.07, 1.87)	1.20 (1.04, 1.58)	0.031*	0.028*	0.575
LDL-C,mmol/L	2.93 (2.16, 3.56)	2.40 (1.99, 2.81)	2.86 (2.36, 3.29)	0.032*	0.857	0.003**
UA, μmol/L	367.0 (264.9, 446)	253.9 (207.9, 301.2)	299.3 (231.9, 381)	0.001***	0.064	0.030*

Data given as median (interquartile range) unless otherwise stated.

HD, hypothalamic dysfunction; BMI, body mass index; WC, waist circumference; IFG, impaired fasting glucose; IGT, impaired glucose tolerance; DM, diabetes mellitus; MetS, metabolic syndrome; ALT, alanine aminotransferase; AST, aspartate aminotransferase; GLU, plasma glucose; TC, total cholesterol; TG, triglycerides; HDL-C, high density lipoprotein cholesterol; LDL-C, low density lipoprotein cholesterol; UA, uric acid. * refers to P<0.05, **, <0.01 and ***, <0.001.

Regarding biochemical profile, the median HDL-C of females with HD was 0.86 mmol/L, which was significantly lower than that of the lean and BMI-matched female control (P = 0.031 and P = 0.028, respectively). No significant difference was noted when comparing HDL-C levels of men with HD to lean and BMI-matched controls. Total cholesterol and triglyceride levels were significantly higher than those in the lean controls (P = 0.004 and P = 0.002, respectively), but no significant difference was noted when compared with BMI-matched controls. Both alanine aminotransferase (ALT) and aspartate aminotransferase (AST) levels in patients with HD were significantly higher than those in the lean control group (P = 0.001 and P < 0.001, respectively), but only AST levels were significantly higher than those in the BMI-matched controls (P = 0.006). The uric acid level of patients with HD was higher than that of the lean and BMI-matched control groups. However, a statistically significant difference was observed only when compared with the lean control group (P < 0.001).

We then analyzed predicators of MetS in patients with HD and BMI-matched controls. **Table 7** presents the result. In univariate analysis, we found that BMI, HD and anterior pituitary dysfunction were significantly correlated with MetS (P=0.001, P=0.030, P=0.020, respectively). Age, sex and CDI were not correlated with MetS. Age, sex and factors significantly correlated with MetS in the univariate analysis were included in multivariate analysis. The result showed that only HD was an important risk factor (P=0.035, OR 2.919, 95%CI (1.077, 7.910)) after adjusted for age, sex and BMI, while anterior pituitary was not considered as an independent risk factor for HD in the multivariate analysis.

**Table 7:** Univariate and multivariate predictors of metabolic syndrome in adults with hypothalamic dysfunction and BMI-matched control.

	Patients with MetS	Patients without MetS	Univariate	Multivariate	
			P value	P value	OR (95% CI)
Demographic characteristics					
Age	32.6 (25.9, 41.0)	30.9 (25.7, 38.3)	0.713	0.737	
Female	18	24	0.722	0.420	
Clinical characteristics					
BMI, kg/m^2^	29.5 (26.5, 34.8)	25.3 (21.6, 29.6)	<0.001	<0.001	1.202 (1.088, 1.329)
Hypothalamic dysfunction	23	21	0.030	0.035	2.919 (1.077, 7.910)
Anterior pituitary dysfunction	20	16	0.020	0.874	
CDI	20	19	0.091	NA	

Data given as median (interquartile range) unless otherwise stated.

MetS, metabolic syndrome; OR, odd ratio; CI, confidence interval; BMI, body mass index; CDI, central diabetes insipidus; NA, not available.MetS, metabolic syndrome; OR, odd ratio; CI, confidence interval; BMI, body mass index; CDI, central diabetes insipidus; NA, not available.

## Discussion

To our knowledge, few studies on adults with HD have been published because HD is far more prevalent in children than that in adults. This study showed a complex clinical picture of HD involving various endocrine and physiological disorders. Considering the demographic characteristics, HD showed a balanced distribution in the two sexes and mainly occurred in middle-aged individuals. Temperature dysregulation, weight gain, and hypersomnia were the common hypothalamic physiological dysfunctions in our study. These symptoms were also frequently observed in a previous case series of children with HD (3). The incidence of anterior pituitary insufficiency (84.7%) was comparable to those previously reported in nonfunctioning pituitary adenoma (65–94%) and craniopharyngioma (40–87%), and higher than that in Langerhans cell histiocytosis (LCH) (50%) (23–25). Notably, the incidence of CDI (81.4%) in our study was higher than that reported in children undergoing HD (3). We found that one-third of our patients with CDI presented with adipsia, which may indicate damage to osmoregulatory and thirst perception pathways comprising the hypothalamic nuclei, namely the median preoptic nucleus (MnPO) and organum-vasculosum lamina terminalis (OVLT) (26, 27). Comprehensive and coherent management approaches are required for ADI, including desmopressin, compensatory fluid intake, daily weighing, and frequent serum sodium monitoring (15, 26).

Neoplasms were the leading cause of HD in our series, among which germ cell tumors and LCH were the most common pathological types, while benign tumors such as craniopharyngioma and pituitary adenoma were less common. Specifically, we report three cases of primary or metastatic non-Hodgkin lymphoma (NHL). Although exceedingly rare, hypothalamic-pituitary involvement in NHL has been reported in a few cases (28–30). Most cases develop hypopituitarism or diabetes insipidus, but hypothalamic physiological dysfunction has rarely been observed (28). In our series, a 55-year-old woman complained of daytime sleepiness, uncontrolled food intake, and personality changes as part of her initial symptoms. MRI, position emission tomography (PET)/CT, and subsequent biopsy confirmed broad intracranial involvement of diffuse large B-cell lymphoma, including hypothalamic infiltration. These cases indicate the importance of a broad differential diagnosis when treating adults with HD. In line with previous reports, our results highlight the importance of screening for HD after surgery in the sellar region, especially masses with an inclination to invade the hypothalamus. While signs of HD develop within a month after surgery, radiosurgery may contribute to a delayed disruption of hypothalamus function up to a year after treatment; thus, close follow-up is required for these patients.

The cause of HD was unclear in six patients. We have considered several rare causes of HD, such as autoimmune hypothalamitis (AHT) and ROHHAD syndrome. MRI of AHT typically revealed an isolated, homogenous hypothalamus mass that significantly enhanced by contrast and the absence of the posterior pituitary bright spot on T1-weighted images (31). The clinical diagnosis of AHT is confirmed by a positive therapeutic response to predinisone. The sellar MRI of these six patients were not consistent with the presentation of hypothalamitis, and none of them showed signs of central hypoventilation, which is essential for the diagnosis of ROHHAD syndrome according to the definition published by Ize-Ludlow in 2007 (32). HD without MRI abnormalities in hypothalamic region has been reported in patients with neural crest tumor (NET), and a systematic review showed that 56% of ROHHAD patients coupled with NET (32, 33). Although no such sign was found in our cases, a screening for tumor is necessary for patients without hypothalamic mass on MRI.

Hypothalamic damage is considered a risk factor for MetS in hypopituitarism of various etiologies (8). The prevalence of MetS (52.5%) in our study was comparable to that reported in some case series of adult-onset craniopharyngiomas ranging from 37–68% (8, 18, 34). We observed a significantly higher risk of MetS in our patients with HD than that in the lean and BMI-matched controls, and HD was proved to be an independent risk factor of MetS in the multivariate analysis after adjusted for age, sex and BMI. The result suggests that besides obesity, many other factors related to HD contribute to the increased risk of MetS in these patients. Unhealthy lifestyles, such as lack of physical activity, overeating and disrupted sleep, may contribute to MetS in these patients. Insulin resistance, chronic inflammation and neurohormonal activation have been regarded as essential players in the development of MetS (35). We found a significantly higher prevalence of elevated fasting glucose than that in the BMI-matched controls. Hypothalamic insulin resistance can result from insulin hypersecretion attributed to upregulation of parasympathetic activity, and previous study suggested that the severity of insulin resistance was apparently influenced by the degree of hypothalamic involvement, regardless of BMI (36, 37). The resulting increase in circulating free fatty acids (FFAs) further leads to increase in cholesterol and TG synthesis, decreased HDL concentration and more visceral fat deposit (35). We also found a significant lower HDL-C level in female with HD. Another important mechanism lies in neurohormonal pathway, namely increased leptin and decreased adiponectin level (35). Previous study has found an elevated leptin level in patients with HO when comparing to obese control, even after adjusting for fat mass (38). This can be attributed to the failure of leptin to act on hypothalamus. Our results indicate that HD is an important risk factor of MetS despite the effect of BMI, probably due to exacerbated glucose metabolism disorder and neurohormonal abnormalities. Therefore, special attention should be paid to the metabolic profile during the follow-up of patients with HD.

Hyperphagia has long been regarded as the major cause of hypothalamic obesity (HyOb). The disruption in essential part for satiety signal in hypothalamus, namely arcuate nucleus (ARC), ventromedial hypothalamus (VMH) and the lateral hypothalamic area, leads to an imbalance in appetite regulating hormone and increased energy intake (2, 39). However, not all patients with HyOb show hyperphagia. In our cohort, hyperphagia was only present in a half of patients who showed weight gain. Recent studies have been attached increasingly importance to decreased energy expenditure in the development of HyOb. Previous studies have reported a decreased energy expenditure in patients with hypothalamic damage regardless of body composition, and this lowered energy expenditure was related with severe clinical HD (40–42). These findings help explain HyOb in our patients without excessive appetite. Measurement of resting energy expenditure can be useful for these patients by helping to determine whether they can benefit from therapy targeting at energy expenditure, such as dexamphetamines (2, 40).

Previous studies have reported a negative effect of hypopituitarism on metabolic profiles. The prevalence of MetS in adult patients with hypopituitarism has increased to 38–43.1% in association with higher cardiovascular and cerebrovascular morbidity (43, 44). Joustra et al. revealed that the number of pituitary axis deficiencies is related to the risk of MetS (45). In this study, we found that panhypopituitarism was a significant risk factor for MetS in patients with HD, proved in both univariate and multivariate analysis. This suggests a greater extent of pituitary hormone deficiency, which might indicate severe hypothalamic damage and lead to an increased risk of MetS. In particular, previous studies found that adult GHD was associated with reduced lean body mass, excess abdominal adiposity, diabetes, and an unfavorable lipid profile, which may serve as a key contributor to the increased risk of MetS in patients with hypopituitarism (44, 45). However, no single axis deficiency, including GHD, was considered as a risk factor of MetS in our cohort. A larger study is needed to clarify whether any single axis deficiency, namely GHD, serves as an independent risk factor for MetS in patients with HD.

Our study has several limitations. First, the sample size was small due to the rare occurrence of HD, which made it difficult to analyze whether HD per se contributed to an unfavorable metabolic profile independent of anterior pituitary hormone deficiency, namely, GHD. In addition, in our health check-up record, we failed to find age-, sex-, and BMI-matched controls for some patients because of their extreme BMI. However, after excluding those with extreme obesity, we still observed a significantly higher risk of MetS in patients with HD, which highlights the prevalence of MetS in these patients. Another limitation was that neuroimaging data were not integrated to assess the size and location of the hypothalamic damage and its effect on the metabolic profile. Furthermore, obesity was measured based on BMI in patients whose WC was unavailable, which may have underestimated the prevalence of abdominal obesity and MetS.

In conclusion, HD is a heterogeneous disease with various causes and clinical symptoms. In the present study, intracranial neoplasms were the most common cause of adult HD. Temperature dysregulation and hypogonadotropic hypogonadism were the most common physiological and endocrine dysfunctions, respectively. In addition, HD was a significant risk factor of MetS after adjusted for BMI. Increased susceptibility to impaired glucose metabolism was found in adults with HD compared with age-, sex-, and BMI-matched controls. Thus, a thorough metabolic evaluation and careful management should be performed in adults with HD.

## Data availability statement

The raw data supporting the conclusions of this article will be made available by the authors, without undue reservation.

## Ethics statement

The studies involving human participants were reviewed and approved by the Ethics Committee of the Peking Union Medical College Hospital (PUMCH). The patients/participants provided their written informed consent to participate in this study. Written informed consent was obtained from the individual(s) for the publication of any potentially identifiable images or data included in this article.

## Author contributions

ZX reviewed the medical records and drafted the manuscript. XK and XY helped to review the medical records. LW, LD, YY, HoY and KD performed clinical management for the patients. FF, XL and HuY helped with analysis of imaging. HP, RW, LL and HZ conceived the research idea and edited the manuscript. All authors contributed to the article and approved the submitted version.

## Conflict of interest

The authors declare that the research was conducted in the absence of any commercial or financial relationships that could be construed as a potential conflict of interest.

## Publisher’s note

All claims expressed in this article are solely those of the authors and do not necessarily represent those of their affiliated organizations, or those of the publisher, the editors and the reviewers. Any product that may be evaluated in this article, or claim that may be made by its manufacturer, is not guaranteed or endorsed by the publisher.
